# Medical and Physical Intelligence

**Published:** 1803-08-01

**Authors:** 


### MEDICAL AND PHYSICAL INTELLIGENCE.

PLAN *of* LECTURES *on the* VACCINE POCK INOCULATION, *at the Institution, (founded Dec.* 1799, *late* No. 5, *Golden-Square,)* No. 44, *Broad-Street, Golden-Square, by* GEORGE PEARSON, M. D. F. R. S. *Physician to the Cow-Pock Institution, &c. &c.*

A Lecture to be given at the Institution once or twice a week, according to the subject of it, for about twelve weeks. The principal objects will be,

1. The history of what is known of the vaccina in cows.

2. The history of the discovery, introduction, and propagation of vaccine inoculation.

3. To shew, in patients at the institution, the progress of the inoculated cow-pock, through its stages of growing into a vesicle, constitutional disorder, scabbing process, deciduary carbuncle-like scab, with a view especially to make known the distinguishing characters of the vaccina.

4. To explain the unusual, or accidental symptoms and effects of the vaccina: viz. eruptions, phlegmonous inflammation, erythema, axillary swellings, essera vaccina, pustule, ulcerations of inoculated parts, &c.

5. To explain the anomalous eruption of inoculated parts.

6. To explain the anamolous course of the inoculated vaccina.

7. Intervening disorders, especially the small-pox, measles, chicken-pox, contagious angina, hooping cough, tooth rash, red gum, &c.

8. In-

8. Instances, by inoculation, of the small-pox and cow-pock at the same time in the same person.

9. The various modes of preserving vaccine matter.

10. The effects of various modes of inoculation.

11. The effects of matter at different ages of the vaccine-pock.

12. The effects of inoculation of persons who have undergone the small-pox, or cow-pock.

13. The effects of inoculation when it fails to destroy the susceptibility of the small-pox.

14. The medical treatment and regimen during the cow-pock.

15. The effects on health subsequently to inoculation.

16. The vaccine inoculation instead of the small-pox, as vicarious of a disease in sheep.

PROPOSALS.

1. Subscribers for life, viz. of ten guineas, to the Vaccine Institution, to be admitted gratuitously; as well as

2. Perpetual pupils to Dr. Pearson's lectures in general; and his

3. Other pupils on becoming perpetual, in addition to their present lectures, to be admitted on the same terms.

4. Those who are neither subscribers to the institution, nor are pupils, as just mentioned, are to pay three guineas for a single course, or six guineas as perpetual.

---

### SMALL-POX AND INOCULATION HOSPITALS, PANCRAS.

*Extract from the Committee's Report presented to an Half-Yearly General Court, held at Batson's Coffee-House, Cornhill, London, on Thursday the* 16*th Day of June*, 1803.

VARIOLOUS INOCULATION.

31,352 patients have received the benefit of this department of the institution from its commencement until the 1st of January 1802; and during the past year only 88 persons have been inoculated for the small-pox, of which number 39 were out-patients; and as only 10 persons have received the variolous inoculation since January last, this practice at the hospital may be considered as generally superseded by the substitution of the

VACCINE INOCULATION.

This practice began in this hospital under the direction of Dr. Woodville, with the assistance of Mr. Wachsell, the resident surgeon, on the 21st of January 1799; and from that period to the 1st of January 1802, the patients received into the hospital

| | |
|---|---|
| were - - - - - - - - - - - - - - - - - - | 1580 |
| Out-patients - - - - - - - - - - - - - - | 5912 |
| During the year 1802, in-patients were - - - - | 337 |
| Out-patients - - - - - - - - - - - - - - | 3990 |
| From the 1st of January 1803, to the 15th of June, instant, in-patients - - - - - - - - - - - | 70 |
| From ditto to ditto, out-patients | 1826 |
| | 13715. |

Of

Of this large number *there has not been any instance in which the inoculation proved unsuccessful.* The committee desire to repeat what they stated in a former report, that 2500 were, after their recovery, found to be secure from the natural smallpox, by receiving the variolous inoculation, without any effect; and that in all this extensive practice, the committee have not heard of any one complaint from amongst those who were not inoculated a second time, of their having taken the natural small pox, although they were chiefly indigent persons, and the far greater number of them living in places where the air is very confined; and particularly where it has been since ascertained, that the natural small-pox was prevalent amongst those with whom many of them necessarily had continual intercourse.

The benefits of this institution, in its respective branches, are not confined to the metropolis or its vicinity; for it has always received patients from considerable distances, and also that applications are continually made to it from various parts of the united kingdom, from America and the West Indies, and also from the British settlements in the East Indies, for correct information of the practice and mode of treatment, and for supplies of matter for inoculation, which have been always readily furnished.

The committee rejoice in being enabled to witness the above numerous instances of its utility, and they invite the continuance of the public support as long only as the poor shall need its refuge and protection.

---

Dr. HEINZE has inoculated more than three thousand sheep with the cow-pock, on the estate of Count N. S. Rumanzoff, in the Ukraine, and thus secured them from a contagious distemper which proved mortal to the flocks of the surrounding districts; and even in the place where the experiment was made, to such of the sheep as had not been inoculated.

---

C. PAYSSE has found, that a lute fit for all the operations of chemistry may be made by mixing eggs, both the whites and yolks, with half their weight of quick lime.

